# Protocol for a Cross-Sectional Study: Effects of a Multiple Sclerosis Relapse Therapy With Methylprednisolone on Offspring Neurocognitive Development and Behavior (MS-Children)

**DOI:** 10.3389/fneur.2022.830057

**Published:** 2022-04-26

**Authors:** Valeska Kozik, Matthias Schwab, Sandra Thiel, Kerstin Hellwig, Florian Rakers, Michelle Dreiling

**Affiliations:** ^1^Department of Neurology, Jena University Hospital, Jena, Germany; ^2^Department of Neurology, St. Josef Hospital, Ruhr University Bochum, Bochum, Germany

**Keywords:** Multiple sclerosis, glucocorticoids, methylprednisolone, cognition, pituitary-adrenal axis

## Abstract

**Introduction:**

Multiple Sclerosis (MS) is the most common neuroimmunological disease in women of childbearing age. Current MS therapy consists of immunomodulatory relapse prevention with disease-modifying therapies (DMTs) and acute relapse therapy with the synthetic glucocorticoid (GC) methylprednisolone (MP). As most DMTs are not approved for use during pregnancy, treatment is usually discontinued, increasing the risk for relapses. While MP therapy during pregnancy is considered relatively save for the fetus, it may be detrimental for later cognitive and neuropsychiatric function. The underlying mechanism is thought to be an epigenetically mediated desensitization of GC receptors, the subsequent increase in stress sensitivity, and a GC-mediated impairment of brain development. The aim of this study is to investigate the associations of fetal MP exposure in the context of MS relapse therapy with later cognitive function, brain development, stress sensitivity, and behavior.

**Methods and Analysis:**

Eighty children aged 8–18 years of mothers with MS will be recruited. Forty children, exposed to GC in *utero* will be compared to 40 children without fetal GC exposure. The intelligence quotient will serve as primary outcome. Secondary outcomes will include attention, motor development, emotional excitability, Attention-Deficit Hyperactivity Disorder-related symptoms, and behavioral difficulties. The Trier Social Stress Test will test stress sensitivity, EEG and MRI will assess functional and structural brain development. To determine underlying mechanisms, DNA methylation of the GC receptor gene and the H19/IGF2 locus and changes in the microbiome and the metabolome will be investigated. Primary and secondary outcomes will be analyzed using linear regression models. Time-variant outcomes of the stress test will be analyzed in two mixed linear models exploring overall activity and change from baseline.

**Ethics and Dissemination:**

This study was approved by the participating institutions' ethics committees and results will be presented in accordance with the STROBE 2007 Statement.

**Trial Registration:**

https://clinicaltrials.gov/ct2/show/NCT04832269?id=ZKSJ0130

## Introduction

### Background

Multiple sclerosis (MS) is one of the most common neurological diseases in young adults, usually occurring between the ages of 20 and 40 years, affecting an estimated 2.8 million patients worldwide (1). Approximately two thirds of these patients are women, mainly of childbearing age (2). Therapy for MS consists of disease-modifying therapy (DMT) for relapse prophylaxis with immunomodulatory substances and relapse therapy with methylprednisolone (MP). As most immunomodulators are not approved for use during pregnancy, treatment is usually discontinued with a subsequent risk increase for relapses (3). While the risk for relapses especially during the first and second trimester is comparable to the prepartum period and thus not an uncommon event, the risk decreases by ~70% during the third trimester (4). Based on these data and the prevalence rate for MS in women of childbearing age (1), we estimate that in Germany ~5,500 MS-relapses occur annually during a pregnancy.

European and US guidelines recommend the use of high-dose MP to treat severe, disabling relapses anytime during pregnancy with a risk-benefit-evaluation between gestational weeks 8 and 11 (5). This treatment is considered relatively harmless for the fetus since no severe physical development disorders have been found at birth (3, 6). However, studies with offspring antenatally exposed to the synthetic GC betamethasone (BM) to enhance fetal lung maturation due to a threat of premature labor suggest a risk for increased stress sensitivity and neuropsychiatric disorders in later life (7, 8). These effects of antenatal GC-exposure on offspring outcomes have been found at all periods of pregnancy (9). Remarkably, the treatment regime used in obstetric indications consists of only 2 × 8–12 mg BM 24 h apart as compared to the 500–1,000 mg MP over 3 – 5 days used to treat an MS relapse. Considering the ~six-fold higher biological activity of BM compared with MP (10), the total dose of GC in the treatment of MS relapses is up to 50-fold higher than for obstetric indications. In a previous study from our workgroup, which is the basis for the present study, we could show that antenatal exposure to 2 × 8 mg BM to enhance fetal lung maturation resulted in multidimensional changes in neurodevelopment and stress-sensitivity in full-term offspring at 8–9 years of age compared to children not exposed to BM (11, 12). This included an intelligence quotient (IQ), which was on average 10 points lower in the exposed group than in the non-exposed group, changes in autonomic activity, attention-deficit hyperactivity disorder (ADHD)-like symptoms and changes in electrocortical activation during a cognitive task. In a follow-up study with 16-year-olds antenatally exposed to BM, we found changes in measures of structural brain development, specifically alterations in gyrification and a reduced cortical thickness in MRI scans (publication in preparation). Combined, these findings have led to a change in German obstetric guidelines already, which now call for a more critical indication for the induction of lung maturation (13, 14).

The adverse long-term effects of antenatal GC exposure may be the result of both a programming of the function of the hypothalamic-pituitary-adrenal axis (HPAA) and maturational effects of GCs on functional and structural brain development. The relationship between adverse environmental influences such as an intrauterine exposure to supraphysiological levels of GCs during critical periods of development and the health of the offspring in later life is the basis of the “Fetal Programming” or “Developmental Origins of Health and Disease (DOHaD)” hypothesis (15, 16). Until the 3^rd^ trimester, the fetus is incapable of synthesizing cortisol and relatively protected from maternal cortisol because the placental enzyme 11ß-HSD 2 inactivates 80–90 % of maternal cortisol to cortisone (17). In contrast to cortisol, most synthetic GCs with the exception of MP are no substrates for 11ß-HSD2 and cross the placenta without inactivation (18). However, even though MP is a substrate for 11ß-HSD 2, high bolus doses of MP as used in MS-relapse therapy rapidly saturate this enzyme and reach the fetus in pharmacological concentrations (6, 19). Once in the fetal circulation, even short-term elevated levels of GCs can permanently change patterns of gene expression and cellular function via epigenetic mechanisms (20). Permanent desensitization of GC receptors involved in negative feedback regulation of the HPAA via DNA methylation of the promoter region of the GC receptor gene *NR3C1* may lead to a life-long hyperactivity of the HPAA and, thus, to increased stress sensitivity in later life (21, 22). Hyperactivity of the HPAA is associated with stress-related neuropsychiatric disorders, including anxiety, depression, and ADHD (21, 22).

Increased levels of GCs induce accelerated tissue differentiation and maturation at the expense of tissue growth (23). While this effect is utilized to induce lung maturation in fetuses at risk of premature birth (24), it occurs in all organ systems and is reflected in a reduced birth weight following antenatal GC treatment (25). Out of all organ systems, the developing brain is particularly vulnerable to elevated GC concentrations due to its complex sequence of precisely coordinated steps of maturation and its intrinsic plasticity (26), a basic property of the brain enabling learning and adaptation. Supraphysiological concentrations of GCs change fetal brain development via antiproliferative effects on neural stem and progenitor cells (27). The subsequent changes in neuronal connections and myelination (28, 29) may contribute to the sustained changes in cognitive and cerebral functioning. Increased concentrations of GCs may also affect fetal brain development indirectly via changes in the composition of the maternal gut microbiota (30, 31), which in turn modulates the fetal gut microbiota (32, 33). The gut microbiome communicates with the central nervous system via an integrated, bidirectional communication pathway which i.a., involves inflammatory cytokines, neuromodulators and neurotransmitters (17, 34, 35). Consequently, disturbances in this communication system, the so-called “gut-brain” axis, have been associated with a number of neurological and psychiatric diseases, such as depression, anxiety disorders, and attention deficit hyperactivity disorder (17, 32, 36, 37).

While no studies examining the long-term effects on the offspring exist, current MS guidelines consider relapse therapy using MP during pregnancy safe for the fetus. Based on findings regarding long-term consequences of antenatal BM exposure, we hypothesize however, that this may not be the case. Based on the up to 50-fold cumulative dose of GCs in MS relapse treatment and its more prolonged administration compared to obstetric indications, we aim to investigate whether relapse treatment with MP in MS patients during pregnancy affects structural and functional brain development, cognition and behavior as well as stress sensitivity in the antenatally exposed offspring.

### Research Aims and Hypotheses

This study is based on two hypotheses: Fetal exposure to MP during MS relapse therapy leads to epigenetically mediated

a. disturbances in functional and structural brain development and
b. a long-term change in stress sensitivity of the offspring.

The resulting changes in brain function are associated with neuropsychiatric abnormalities as well as cognitive and behavioral changes in later life.

The primary objective of this cross-sectional study is to elucidate associations of antenatal MP exposure with cognitive performance in children aged 8–18 years compared to non-exposed children of mothers with MS. As secondary objectives, this study will investigate the association of cognitive development and behavioral problems with underlying changes in stress sensitivity and functional and structural brain development. To clarify potential epigenetic mechanisms underpinning these changes in stress sensitivity and functional and structural brain development, DNA methylation at the promotor region of the GC receptor gene *NR3C1* and at the H19/IGF2 locus, which controls expression of the insulin-like growth factor 2 (IGF2), the major growth hormone during development (38), will be investigated. To elucidate further potential mechanisms, changes in the composition of the microbiome will be examined.

This study is expected to advance the understanding of developmental programming effects of antenatal high-dosage GC exposure. Another major aim of this study is to add data to the debate around evidence-based decision-making regarding (1) the continuation of a DMT during pregnancy *vs*. the potential long-term risks of relapse therapy with MP for the child's cognitive and neuropsychiatric health in later life, and (2) the risks of relapse treatment with MP for the child *vs*. the risks of an untreated relapse for the mother. There is an accumulating amount of *real world* data on the safety of continuing a DMT during pregnancy primarily with interferons or glatiramer acetate, which may be safer for mother and child than a potentially necessary relapse treatment with MP.

## Methods and Analysis

### Study Design

This two-center, observational, cross-sectional study aims to recruit *N* = 80 children of mothers with MS. The exposed group (*n* = 40) will consist of children who were antenatally exposed to MP as part of maternal MS relapse therapy, while the control group (*n* = 40), will consist of children of mothers with MS, who were not exposed to MP. Assessment will take place at the University Hospital in Jena (UKJ) and the Ruhr University Bochum (RUB). The first participant was included on 19/10/2020 and the study is anticipated to continue until the end of 2024.

### Sample Size

The aforementioned study by some of the authors (11) investigated cognitive performance, as measured by an IQ test, in two groups of 39 healthy children at the age of 8 years. Considering the lower biological activity of MP compared to BM, the cohort in the present study was exposed to a GC-dose up to 50-fold higher than in the study on the effects of BM. The higher dosage of MP will most likely expose a significant effect in two groups of the same size as the previous study.

As the present study uses a different IQ test, the planned sample size is further based on the standardized effect size Cohen's *d*, as a multiple of the standard deviation. The previous study found a mean difference in IQ score of 10.5 points (SD = 14.0). A two-sample *t*-test (α = 0.05) would have sufficient power of 82% (*d* = 0.71) to 87% (*d* = 0.75) with two groups of 35 children. This takes into account potential non-evaluability of five participants per group, e.g., in the case of artifacts or attrition. The planned regression models include seven variables, which, based on the *rule of ten* leads to a sufficient number of cases to produce robust results (39).

### Sample Characteristics and Recruitment

Children aged 8–18 years, of any sex and ethnicity are eligible to participate. Based on the children we identified for the group exposed to GC, control children are matched 1:1 by age and sex. A physician presents verbal and written information about the study, its aims, the procedure, and data management to the participants and their guardian(s) and answers any remaining questions. Children are included if they speak German and they and their guardian(s) are willing and able to consent to their participation.

Exclusion criteria for both groups comprise preterm birth (before 34^th^ week of gestation), a birth weight below the 5^th^ percentile, and perinatal complications, such as cerebral hemorrhage, neonatal intensive care requiring ventilation, or any further antenatal therapy with GCs beyond MS relapse therapy. Participants are further excluded from the study in case of maternal substance abuse during pregnancy or serious illness in the child which makes assessment impossible, such as an intellectual disability, or long-term medication with GCs, e.g., in asthma.

Recruitment takes place in the UKJ's close cooperation with the RUB, the host of the *German MS and Pregnancy Registry*, and several other MS centers across Germany. A search algorithm was developed to identify eligible participants for the MP-exposed and the control group within the respective hospital-specific data management systems. Further recruitment efforts are directed toward online advertisements on MS-specific websites and UKJ's public outreach channels.

### Outcome Measures

Basic sociodemographic and medical data related to the pregnancy, birth and the mother's MS are collected from all participants (see Table 1). Combined with a questionnaire on stressful life events during pregnancy, this serves as a proxy for the mother's stress level during pregnancy. The child's current overall stress level is estimated using a questionnaire on stressful life events in the past 12 months (see Table 1). Time between GC exposure and assessment will be calculated using the child's age and the pregnancy week at the time of treatment. The primary outcome measure of this study is the child's global cognitive ability, as measured by a standardized IQ-test, i.e., the Reynolds Intellectual Assessment Scales and Screening (RIAS) (40). Secondary behavioral outcomes, such as motor development, attention, emotional excitability, ADHD-associated symptoms, and behavioral difficulties in multiple categories are measured via neuropsychological assessment, and self-, and informant-reported questionnaires. Additional secondary outcomes are obtained during the Trier Social Stress Test for children (TSST-C), an established standardized protocol to test stress sensitivity (41). These outcome measures comprise of the neuroendocrine (salivary cortisol) and autonomic stress response (salivary alpha-amylase concentration as a measure of sympathetic activity (42)) and heart rate variability (HRV) indices. To capture subjective stress levels during the TSST-C, the participant fills out an anxiety questionnaire (43) before and after the test. As measures of neurodevelopment, activation in the EEG during rest and a cognitive task included in the TSST-C and MRI-based markers of structural brain development are used. With the use of peripheral blood (leukocytes), and buccal mucosa cells, potential epigenetic mechanisms underlying changes in cognition and behavior as well as in stress sensitivity will be determined (see Table 2). For exploratory mechanistic analysis, the composition of the microbiome (stool sample), metabolome (EDTA plasma) and elementome (hair sample) will be examined.

**Table 1:** Basic clinical and demographic data collected.

**Variables**	**Instruments/scale**
Demographic data of participant and guardian(s)	Demographic and socioeconomic data are collected from the participants and their guardian(s) with questionnaires and an interview, including age, sex, height, weight, guardians' education (highest degree), marital status, and chronic health conditions. Collected on NPT day.
Basic pregnancy and birth data	Pregnancy week at time of relapse and treatment is recorded for children exposed to GC. Birth data for the child (gestational age at birth, birth weight and length, head circumference at birth) and mother (diabetes mellitus and pre-eclampsia) are collected via an interview and the consultation of medical files. Collected on NPT day.
MS-specific medical data	To describe the mother's MS status during pregnancy, medical files and the mother are consulted regarding disease course and duration, therapies, and disease progression. Collected on NPT day.
Stressors during pregnancy (mother)	Based on similar validated instruments, a questionnaire adapted to this study's aims is used to assess stress during the pregnancy. For each trimester, the mother indicates any stressors related to personal, familial, or work life, or noxious substances intake. Collected on NPT day.
Recent stressors (child)	A German self-report questionnaire is used to determine stressful life events in the past 12 months of the child's life (Zürcher Life Events List) (44), involving home life, school, friendships, etc. In the affirmative case, the participant rates the level of stress the event caused on a 5-point scale. Collected on NPT day.

*NPT, Neuropsychological Testing*.

**Table 2:** Primary and secondary outcome measures regarding the child.

**Variables**	**Instruments/scale**	**Expected outcome**
**Primary outcome measure**	
Intelligence quotient (IQ)	General cognitive ability is examined using the German version of the Reynolds Intellectual Assessment Scales and Screening (RIAS) (40). It examines facets of verbal and nonverbal intelligence and memory capacity. Collected on NPT day.	Lower IQ in GC-exposed children vs. control children
**Secondary outcome measures**	
Cognitive and behavioral performance	
Selective attention, vigilance, impulsivity	A continuous performance test (CPT) (45) is used to assess attentional performance in terms of selective attention, vigilance, and impulsivity. Collected on NPT day.	Worse performance in GC-exposed vs. control children
Motor development	To examine motor development, the Movement Assessment Battery for Children – Second Edition (M-ABC-2) (46) is used, which assesses the developmental adequacy of manual dexterity, ball skills, and balance. Collected on NPT day.	Worse motor development in GC-exposed vs. control children
Emotional excitability	To assess emotional excitability, the corresponding scale of a German self-report personality assessment for children (47) is used. Collected on NPT day.	Higher emotional excitability in GC-exposed vs. control children
Attention deficit disorder symptoms (attention, hyperactivity, and impulsivity)	A German parent-reported questionnaire is used to assess attention deficit hyperactivity disorder associated behavior (*FBB-ADHS* from *DISYPS-III*) (48). Collected on NPT day.	More or more-severe ADHD symptoms in GC-exposed vs. control children
Behavioral difficulties I	The German version of the parent-reported Child Behavior Checklist for children (*CBCL/6-18R*) (49) is used to examine behavioral symptoms on eight scales - (1) anxious/depressed, (2) withdrawn/depressed, (3) somatic complaints, (4) social problems, (5) thought, sleep, and repetitive behavior problems, (6) attention problems, (7) rule-breaking behavior, and (8) aggressive behavior. Collected on NPT day.	More or worse behavioral difficulties in GC-exposed vs. control children
Behavioral difficulties II	A parent-reported screening questionnaire, the German version of the Strengths and Difficulties Questionnaire (50) is used to assess emotional and behavioral problems, hyperactivity and inattention, peer relationship problems, and prosocial behavior. Collected on NPT day.	More or worse behavioral difficulties in GC-exposed vs. control children
Stress reactivity during the Trier Social Stress Test for children (TSST-C)		
Neuroendocrine stress response: salivary cortisol concentration	To determine HPAA activity at rest and during stress, a total of 9 saliva samples are collected throughout the TSST-C and at home (see Figure 2). Concentration of salivary cortisol will be estimated using a commercially available chemiluminescence immunoassay. Collected on NPT day and at home.	Higher HPAA activity in GC-exposed vs. control children
Autonomic stress response (ANS): salivary alpha-amylase concentration	The same 9 saliva samples are used to determine salivary alpha-amylase concentrations (marker for sympathetic activity) at rest and during stress. Concentrations will be estimated by an enzymatic colorimetric assay with an autoanalyzer. Collected on NPT day and at home.	Higher sympathetic activity in GC-exposed vs. control children
Autonomic stress response: heart rate variability (HRV)	Throughout the TSST-C, the participant's heart rate is recorded continuously using ECG (bipolar precordial Nehb lead). HRV indices will include linear and non-linear parameters. Collected on NPT day.	Shift toward a higher sympathetic tone in GC-exposed vs. control children
Subjective stress level	A German anxiety questionnaire (Kinder-Angst-Test-III) (43) is used to measure subjective levels of anxiety before and after the TSST-C (see Figure 2). Collected on NPT day.	Exploratory
Functional brain development	
Electrocortical activity	EEG recording is carried out continuously throughout the TSST-C, with electrodes placed on a standard cap according to the international 10–20 system. The activation patterns are evaluated using spectral power analysis (i.a. spectral edge frequency) and non-linear methods (12, 51). Collected on NPT day.	Higher spectral edge frequency in GC-exposed vs. control children
Structural brain development	
Structural *brain age*	The structural brain age is estimated with the help of the *BrainAGE Score* (52), which is based on a volumetric T1-MRI standard sequence. The *BrainAGE* score is a morphometric parameter that is derived from multivariate voxel-wise analyses based on a machine learning approach. Using this technique, the structural brain age is estimated with 1.5 years precision. Additional markers are gyrification and cortical thickness. Collected on MRI day.	Exploratory
Anatomical connectivity	Diffusion tensor imaging is used to examine complex structural networks that form the connectome, with a focus on regions particularly relevant to stress sensitivity, such as the limbic system including amygdala and hippocampus (29). Collected on MRI day.	Exploratory
Mechanisms underlying changes in cognition, behavior, and stress sensitivity		
Epigenetic mechanisms	Genome-wide and targeted candidate gene analysis of DNA methylation using advanced sequencing techniques (Illumina) on genes involved in the stress system and brain development (e.g., *NR3C1* and IGF2/H19 locus) (20). Material: peripheral blood (leukocytes) and buccal mucosa cells. Collected on NPT day.	Exploratory
Microbiome	Microbiota composition will be analyzed using high-throughput DNA sequencing techniques with fecal specimens collected with the Omnigene Gut OM-200 stool collector. Collected at home.	Exploratory
Metabolome	High throughput gene-sequencing (HTS) to explore potential links between antenatal GC exposure and the composition of the metabolome (17). Material: EDTA plasma. Collected on NPT day.	Exploratory
Elementome	Hair trace elementary profile analysis (*elementomics*) via inductively coupled plasma mass spectrometry to explore 47 mineral and toxic markers (53). Collected on NPT day.	Exploratory

*NPT, Neuropsychological Testing, GC, Glucocorticoid*.

### Procedure

**Figure 1:**
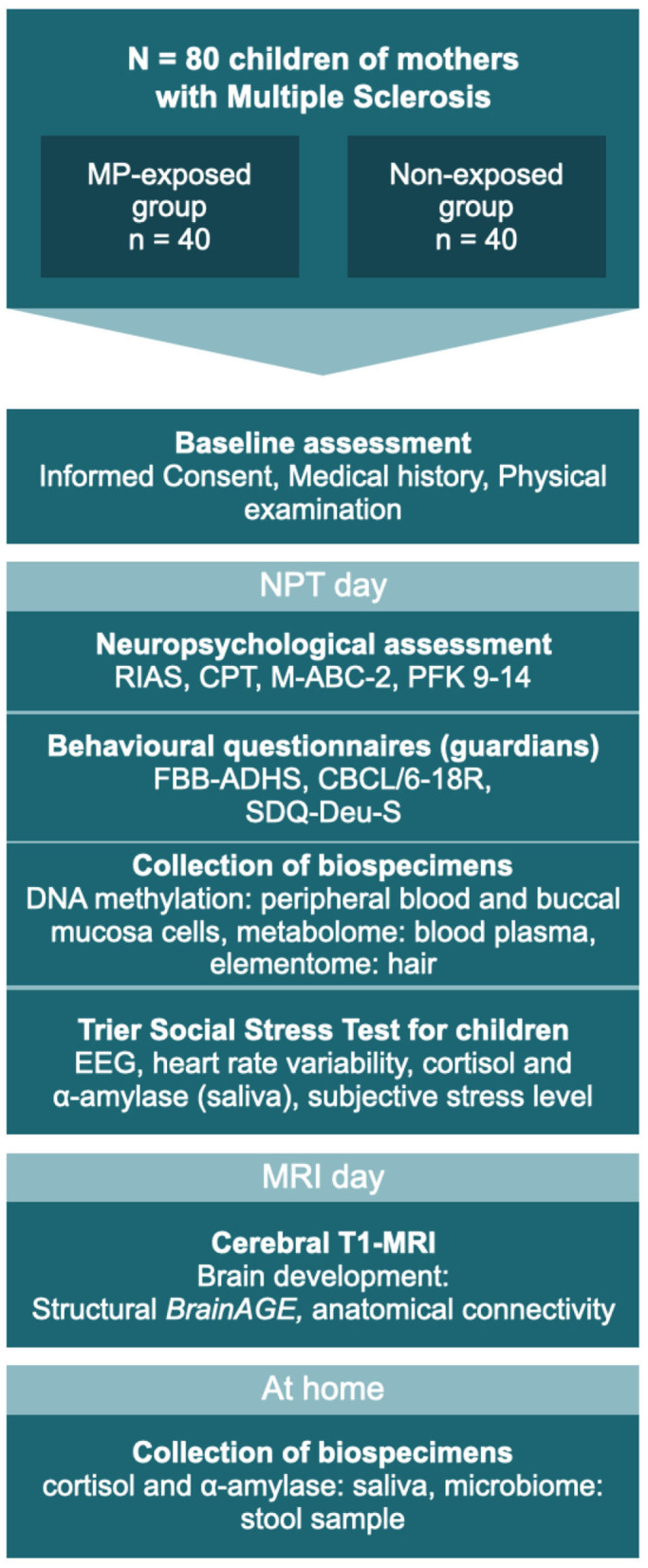
The two study days: testing sequence for all participants. MP, methylprednisolone; NPT, Neuropsychological testing; RIAS, Reynolds Intellectual Assessment Scales and Screening; CPT, Continuous Performance Test; M-ABC-2, Movement Assessment Battery for Children – Second Edition; PFK 9-14, German personality questionnaire; FBB-ADHS, German questionnaire for attention deficit hyperactivity disorder; CBCL/6-18, Child Behavior Checklist for children aged 6–18 years; SDQ-Deu-S, Strengths and Difficulties Questionnaire. MRI and NPT days may occur in reverse order.

Based on logistical considerations, eligible participants are invited to two study days (see Figure 1). On one of the days, neuropsychological testing (NPT) and the TSST-C take place (NPT day), while an MRI scan is carried out on the other (MRI day). For scheduling reasons, a few days may lie in between visits.

On the NPT day, basic clinical and demographic data are recorded and the accompanying guardian is asked to assess their child's behavior using several well-established questionnaires (see Tables 1, 2). The participating child takes part in a set of four neuropsychological assessments of cognition and behavior (see Table 2), performed by a psychologist blinded to the participant's status of antenatal MP exposition. Before the participant is released for a 60-min break, hair, saliva, and buccal specimens are collected. The entire procedure takes ~120–180 min.

**Figure 2:**
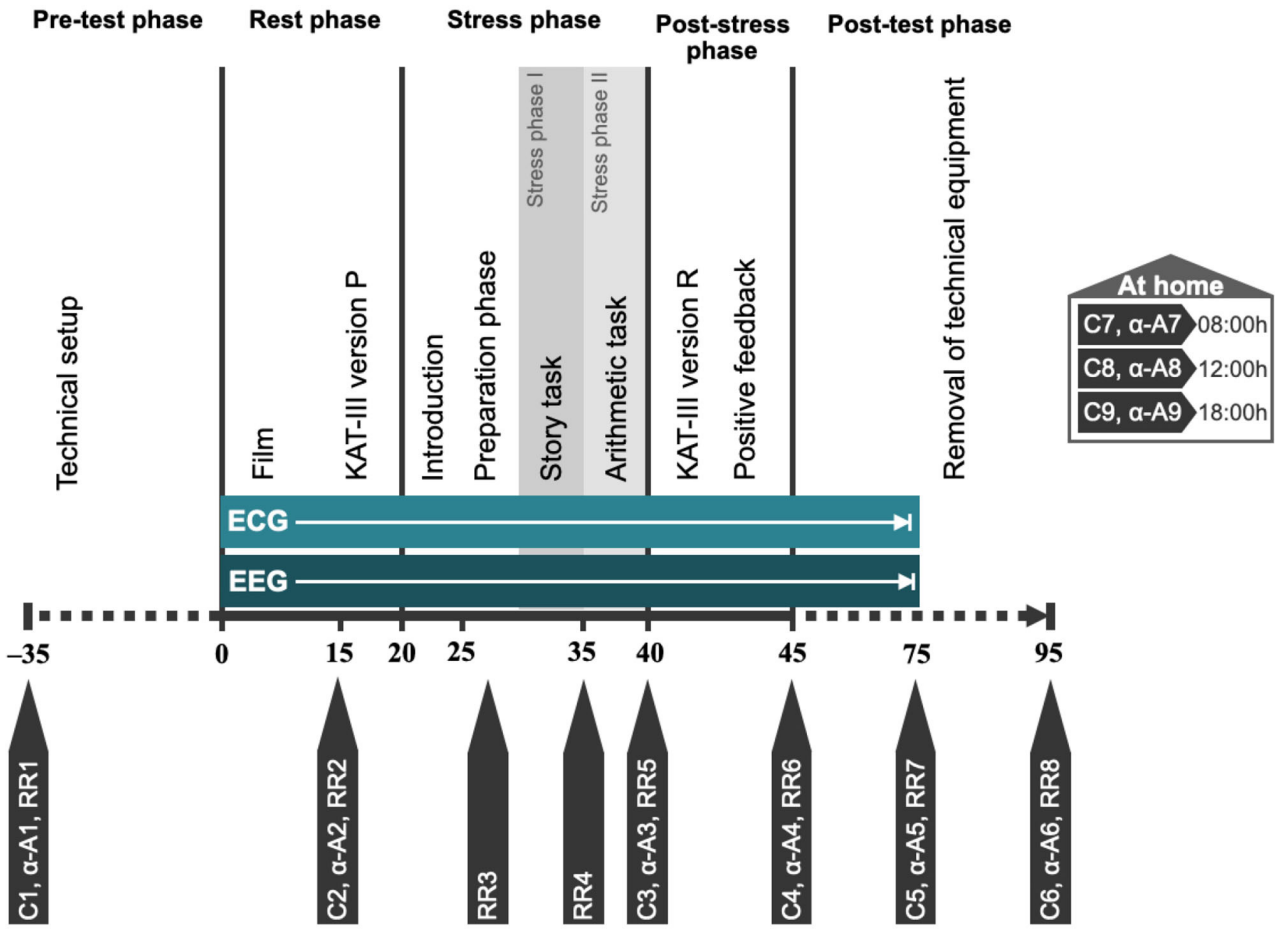
Schematic representation of TSST-C procedure. C1–C9, saliva swabs 1–9 (cortisol); α-A1–α-A9, saliva swabs 1–9 (alpha-amylase); RR1–RR8, blood pressure measurements 1–8; KAT-III, German anxiety scale for children (Kinder-Angst-Test-III) – versions P (prospective) and R (retrospective).

Following the collection of blood (see Table 2), stress sensitivity is examined using the TSST-C, an instrument to generate a psychophysiological stress response in the standardized setting of a simulated examination. See Figure 2 for a schematic representation of the procedure. The participant is equipped with EEG and ECG electrodes, a blood pressure monitor, and a pulse oximeter for continuous measurements during the TSST-C. Saliva samples are collected to measure cortisol and alpha-amylase concentrations throughout the test (see Figure 2 and Table 2 for scheduling of sampling). The TSST-C begins with an “active rest” phase of 15 min, during which the participant watches a slow-paced animal documentary, sitting down. In a separate room, a two-person examination committee then present the participant with a story, before leaving the room for 5 min, giving the participant time to prepare an “exciting and compelling” ending to the story. The participant is then asked to continue telling the story (stress phase I), after which the participant is instructed to verbalize serial subtractions of age-appropriate difficulty (stress phase II). When the participant makes a mistake, the examiner interrupts them with a prompt to start over. The stress phases last a combined 10 min, after which the participant receives positive feedback from the committee. In total, the TSST-C takes ~130 min. After the TSST-C, the participants are handed a kit for the at-home collection of a further 3 saliva samples, to capture the HPAA activity throughout a normal day. The kit also includes material to collect a stool sample for the analysis of the microbiome.

On the MRI day, a volumetric cranial MRI scan in T1 standard sequence is obtained from the participant. Diffusion tensor imaging (DTI) is used to determine the anatomical connectome. Including participant education, the entire procedure is estimated to take 50 min.

### Data Storage and Management

All data are recorded in a pseudonymised form. In accordance with EU data protection legislation, both study centers maintain a secured patient identification list with full names, addresses, and dates of birth. Any personal information is archived for at least 10 years after inclusion of the last participant.

The collection, management and use of human biological specimens in this study is in accordance with national and EU legislation. Any transfer of specimens between the two centers will take place in accordance with the Oviedo Convention (Convention on Human Rights and Biomedicine – CETS 164) and Recommendation of the Committee of Ministers to Member States on research on biological materials of human origin (adopted by the Committee of Ministers on 15 March 2006 at the 958th Meeting of Ministerial Deputies). Due to the vulnerable nature of a cohort of children, we further implement directive 95/46/EC and working group 8/2010 opinion, article 29 and take the high importance of data protection in vulnerable populations into careful consideration.

Part of the initial data collection uses hard copy files, which are stored in a secure location and later digitized using an electronic case report form. All digitized data are stored on a safe server managed by the Center for Clinical Studies at the University hospital in Jena.

### Data Analysis Plan

For the analysis of the primary outcome, exposed and non-exposed groups will be compared in terms of their performance on the IQ test using a linear regression model taking into account potential confounders, including antenatal exposure to MP, point in time of exposure, maternal age at birth, birth weight, and socioeconomic background. Exploratory analysis of secondary outcomes will be performed with a linear regression model. Those outcomes measured repeatedly throughout the course of the TSST-C will be evaluated as time series data with a linear mixed model, with time as a further independent factor. For this analysis, two models will be fitted to explore (1) overall mean activity throughout the TSST-C, as well as (2) the change from baseline, in which the stress response will be adjusted to the baseline value.

### Methodological Challenges

Recruitment of eligible participants presents the main challenge for this study. However, based on estimations of the prevalence of MS in Germany as well as pregnancy and relapse rates, our sample size represents only ~0.8% of the total target population in Germany. The close cooperation with the *German MS and Pregnancy Registry*, founded by members of the study group, gives us access to a database of more than 3,000 pregnancies of women with MS. Several MS clinics and surgeries across Germany, as well as the German Society for MS (DMSG) support recruitment efforts by sharing flyers and online adverts with their patients or members, and searching their databases for potential participants. The ongoing COVID-19 pandemic presents another challenge which slowed down recruitment and testing, particularly in the first quarter of 2021. In accordance with the rules and recommendations of the German government, we have implemented a strict hygiene plan for participants and researchers to continue with recruitment.

The TSST-C is vulnerable to subjective factors in the examination committee and environmental factors. The procedure is guided by detailed protocols and pilot runs, to ensure standardization within and between study centers. The same investigator performs neuropsychological testing in both study centers, granting further consistency of conditions.

To account for the vulnerability of the study population and potential anxiety around measures such as MRI or blood sampling, we devised detailed, child-friendly participant education material. The children's guardians are usually present and the child is offered a topical local anesthetic in preparation for the drawing of blood.

In this study, antenatal or postnatal stressors may act as confounders. Maternal psychosocial stress during pregnancy has been shown to elicit increased maternal and fetal cortisol levels (54, 55). It is further associated with an increase in the offspring stress response, disturbed motor and structural brain development and an increased risk of cognitive, behavioral, and emotional problems in later life, such as autism spectrum disorders, ADHD, depression and schizophrenia (for reviews see (9, 55)). MS relapses are not only treated with GCs but also are a major psychological stressor. However, as relapses during pregnancy rarely go untreated, there is no realistic way of mitigating this confounder using an untreated MS control group. Due to the retrospective nature of this study, there is no reliable method to assess perceived psychological stress level beyond major stressful life events, at a minimum of 8 years in the past. The mother's stress level during pregnancy is thus estimated using a questionnaire based on the Stress and Adversity Inventory (STRAIN) (56), which was adapted to this study's context. Additionally, the children fill out a questionnaire regarding subjective and objective stress in the past 12 months (see Table 1).

## Ethics and Dissemination

This study is conducted with the approval of the ethics committees of Friedrich-Schiller University Jena (reference: 2020-1668-3-BO) and the medical faculty of Ruhr University Bochum (reference: 21-7192 BR). The results are going to be published in relevant peer-reviewed journals, presented in accordance with the STROBE 2007 Statement (57).

## Author Contributions

VK: writing of the manuscript, study implementation, and data collection. MS: study conception and design and critical manuscript revision. ST and KH: critical manuscript revision and study implementation. FR: study conception, design, study implementation, and critical manuscript revision. MD: study conception, design, critical manuscript revision, study implementation, and data collection.

## Funding

This study is supported by the Grant for Multiple Sclerosis Innovation 2020 (MerckHealthcare KGaA). The funder was not involved in the study design, collection, analysis, interpretation of data, the writing of this article or the decision to submit it for publication.

## Conflict of Interest

The authors declare that the research was conducted in the absence of any commercial or financial relationships that could be construed as a potential conflict of interest.

## Publisher's Note

All claims expressed in this article are solely those of the authors and do not necessarily represent those of their affiliated organizations, or those of the publisher, the editors and the reviewers. Any product that may be evaluated in this article, or claim that may be made by its manufacturer, is not guaranteed or endorsed by the publisher.
