# Erratum: Protocol for a cross-sectional study: effects of a multiple sclerosis relapse therapy with methylprednisolone on offspring neurocognitive development and behavior (MS-children)

**DOI:** 10.3389/fneur.2023.1213956

**Published:** 2023-05-15

**Authors:** Frontiers Production Office

**Affiliations:** Frontiers Media SA, Lausanne, Switzerland

**Keywords:** multiple sclerosis, glucocorticoids, methylprednisolone, cognition, pituitary-adrenal axis

Due to a production error, the reference for “11” was incorrectly written as “Li C, Kaiyu L, Sooranna SR, Bennett PR., Zinging L, Dimitris G, et al. The effect of cAMP on PRE promoter activity in presence or absence of progesterone. In: *57th Annual Meeting of the Society for Gynecologic Investigation*, Vol.17. Orlando, FL: Reproductive Sciences (2010). doi: 10.1177/193371912010173s067”. It should be “Rakers F, Schleußner E, Muth I, Hoyer D, Rupprecht S, Schiecke K, et al. Impact of antenatal glucocorticoid exposure on activity of the stress system, cognition and behavior in 8–9-year-old children: a clinical cohort study. (in preparation).”

The publisher apologizes for this mistake. The original article has been updated.

